# 755. Infectious Diseases Journal Watch: a Pilot Study

**DOI:** 10.1093/ofid/ofad500.816

**Published:** 2023-11-27

**Authors:** Benjamin Chen, Darcy Wooten

**Affiliations:** University of California San Diego, San Diego, California; University of California, San Diego, San Diego, CA

## Abstract

**Background:**

Staying apprised of the scientific literature can be a challenge for health science researchers and clinical practitioners alike [1, 2]. Journal surveillance is a popular modality for disseminating curated topic-specific health science research in a scheduled newsletter. The *New England Journal of Medicine* (NEJM) Journal Watch is a prominent example of this format [3], but despite its wide adoption, little research has examined the effectiveness of this model as an educational tool.

**Methods:**

We conducted a pilot study examining the impact of journal surveillance as an educational tool within the University of California San Diego’s (UCSD) Division of Infectious Diseases. Modeled after the NEJM Journal Watch, we created an internal monthly newsletter summarizing the latest research in the field of infectious diseases (ID). We then surveyed readers to evaluate the content covered in article summaries, the barriers and facilitators to learning from the newsletter, and the impact on self-reported clinical knowledge and practice.

**Results:**

Across 10 monthly editions we summarized 45 articles covering a diverse array of content topics published in high impact journals (Figure 1). Thirty-two (71.1%) summaries included an infographic or visual abstract. Eighteen ID division members completed the survey evaluation of the monthly newsletter; most were fellow physicians or early career faculty (Figure 2). Thirteen respondents (71.1%) had read 4 or more editions. Most (94.4%) viewed ID Journal Watch favorably, citing the ability to remain up to date with literature and to improve clinical care after reading the newsletter (Figure 3). Eleven (61.1%) reported that their main barrier to engaging with the newsletter was a lack of time. Infographics were viewed favorably by a majority (66.7%) as a learning tool, supporting previous findings that visual media can decrease cognitive load when interpreting new information [4].

Figure 1

**Figure:**
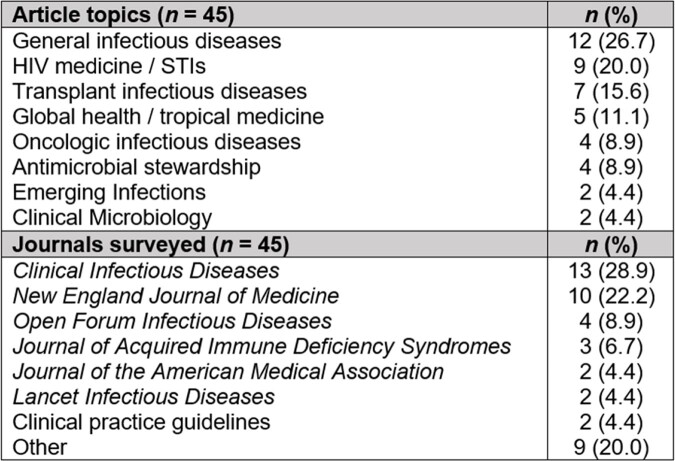

Article topics and journals surveyed from 45 article summaries

Figure 2

**Figure:**
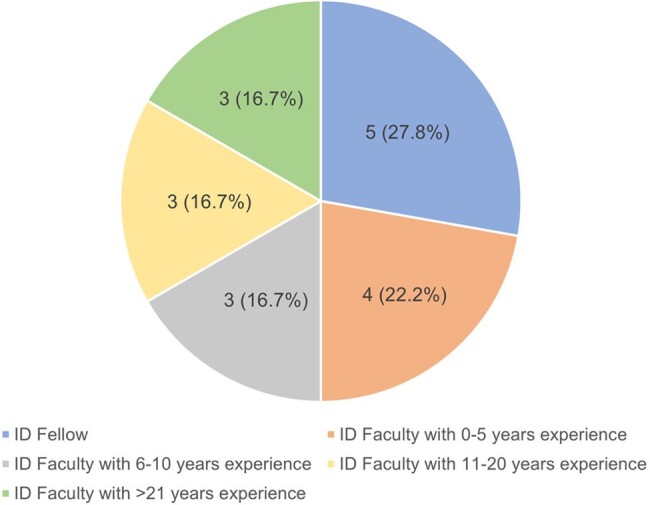

Demographics of survey respondents, n = 18 (%)

Figure 3

**Figure:**
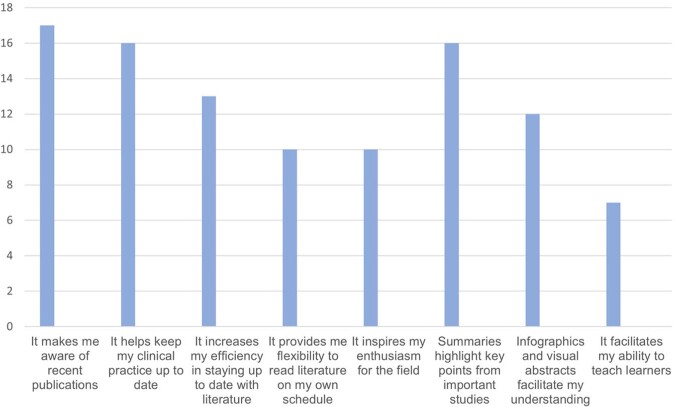

Self-reported benefits of engaging with ID Journal Watch, n = 18

**Conclusion:**

Journal surveillance is a valuable learning tool particularly for trainees and early career faculty. The format allows for rapid acquisition of knowledge that is relevant to clinical care. Visual media should remain a key component of journal surveillance due to its ability to facilitate comprehension.

**Disclosures:**

**All Authors**: No reported disclosures

